# Supplementary material for: Local Climate Adaptation in Chinese Indigenous Pig Genomes
Source: Animals (Basel). 2025 Aug 18;15(16):2412. doi: 10.3390/ani15162412 (PMC12382954; doi:10.3390/ani15162412)
Supplement: Supplementary file 1 [file animals-15-02412-s001.zip › animals-3782206-supplementary-final.pdf]

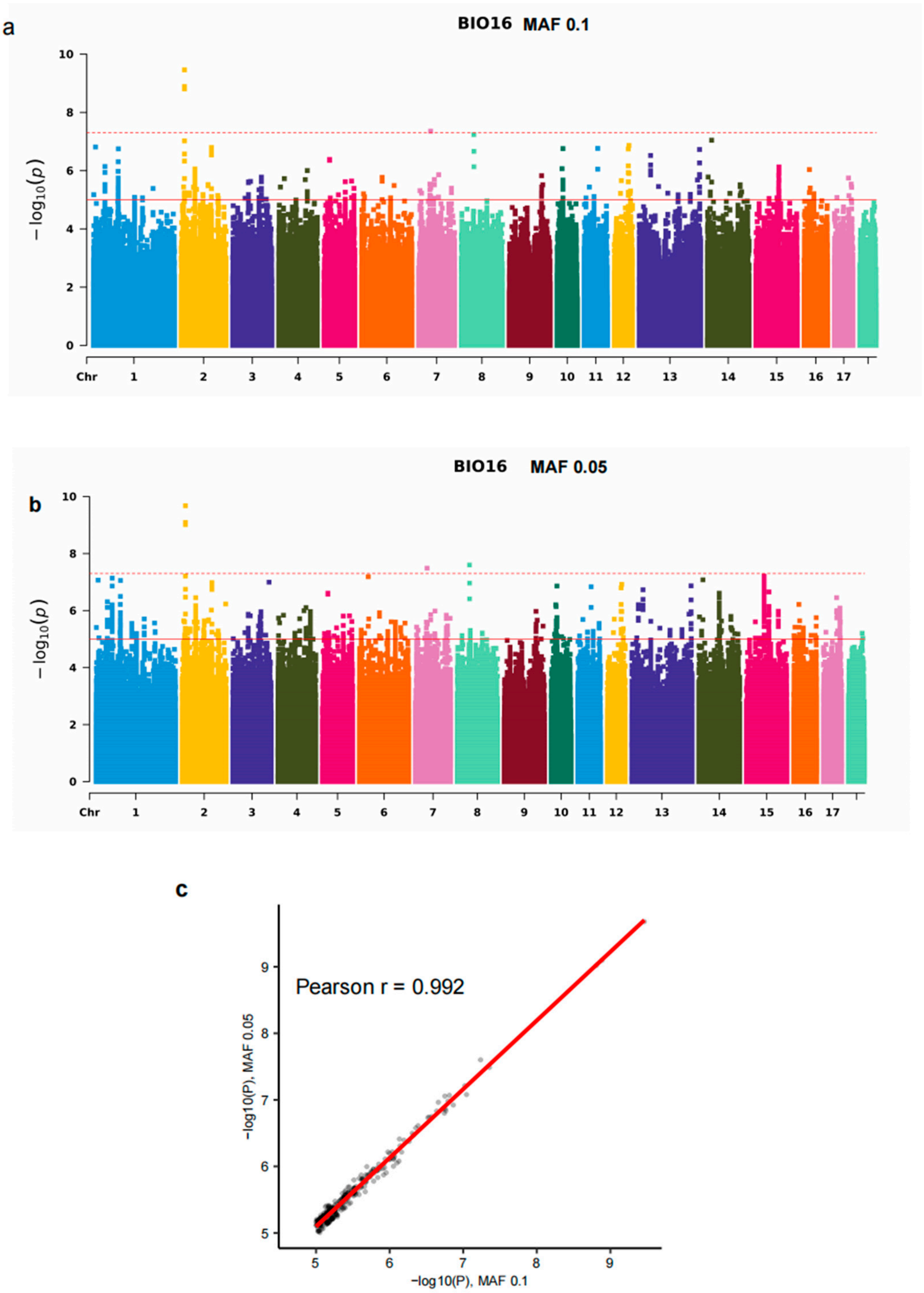

Supplementary Figure S1. Robustness of genotype–environment association (GEA) results under different minor allele frequency (MAF) thresholds for BIO16 (precipitation in the wettest quarter). (a) Manhattan plot of LFMM association results using a MAF > 0.1 filter. (b) Manhattan plot of LFMM association results using a more lenient MAF > 0.05 filter. (c) Scatterplot comparing -log10(P) values from the two analyses. The red line indicates the identity line. The Pearson correlation coefficient (r = 0.992) indicates near-complete concordance between the two MAF thresholds.

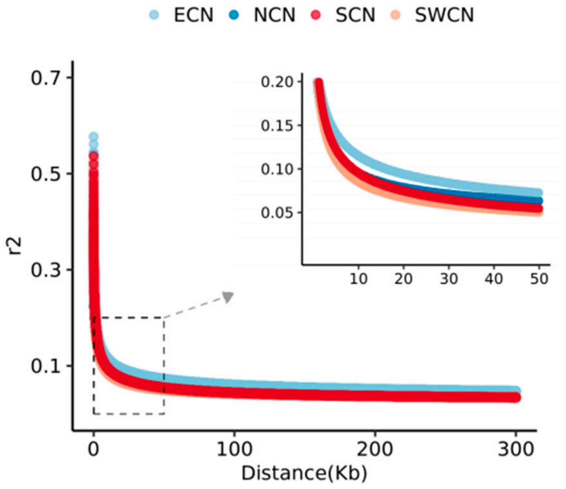

Supplementary Figure S2. Genome-wide linkage disequilibrium (LD) decay across four Chinese indigenous pig population groups. The LD measure (r^2) was plotted against the physical distance between SNPs (in kilobases, kb) for each group: ECN (East China), NCN (North Central China), SCN (South China), and SWCN (Southwest China). The inset zooms in on the 0–50 Kb range, showing that LD rapidly decays and reaches a plateau around 40–50 Kb, which was used to justify the ±25 Kb window for candidate gene identification.

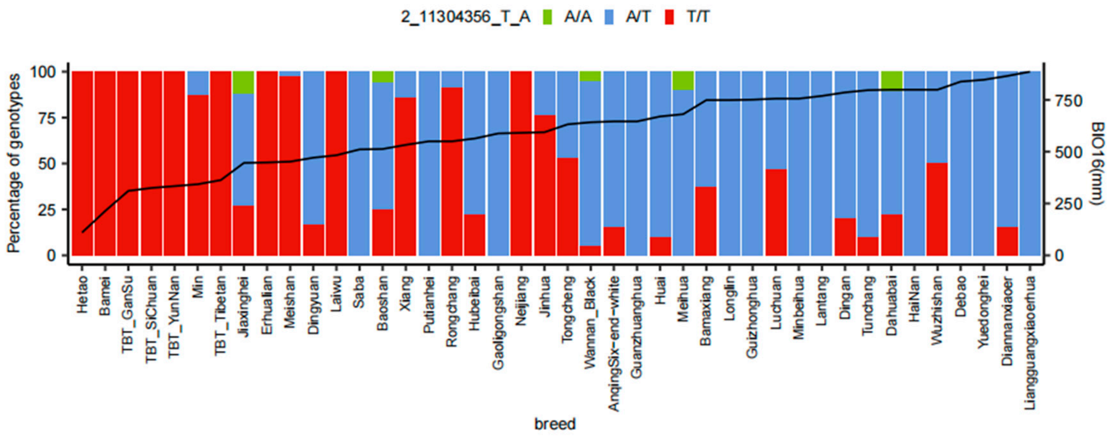

Supplementary Figure S3. Genotype frequency distribution of the top BIO16-associated SNP (2_11304356_T_A) across 46 Chinese indigenous pig breeds.

Bar plots represent the proportion of each genotype within a breed (A/A in green, A/T in blue, T/T in red), and the black line indicates the BIO16 value (precipitation in the wettest quarter) of the corresponding breed's sampling region. The pattern suggests a higher frequency of heterozygotes (A/T) in breeds from humid regions and a predominance of the T/T genotype in breeds from drier regions, supporting genotype–environment associations.

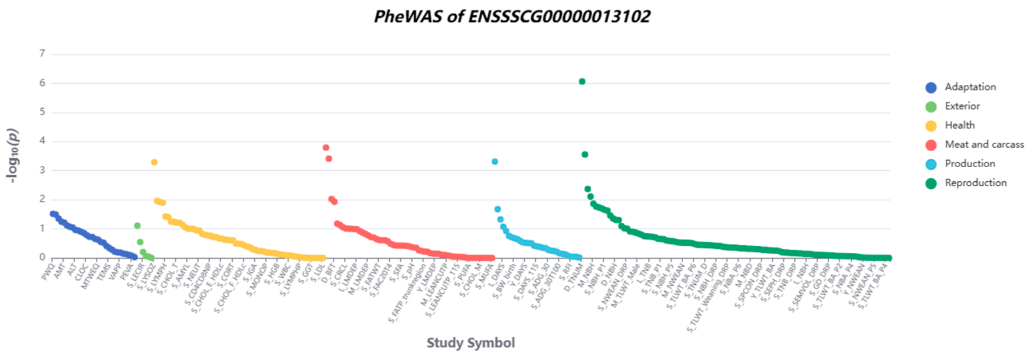

Supplementary Figure S4. Phenome-wide association analysis (PheWAS) of the candidate gene MS4A7 (ENSSSCG00000013102) based on PigBioBank data.

Each dot represents an association between *MS4A7* and a pig trait category, with trait categories color-coded: adaptation (blue), exterior (yellow), health (green), meat and carcass (red), production (cyan), and reproduction (dark green). The y-axis shows the significance level as $-\log_{10}(p)$. The gene shows trait associations across multiple biological systems, notably reproduction and meat-related traits.
